# *Bumba*, a replacement name for *Maraca* Pérez-Miles, 2005 and *Bumba lennoni*, a new tarantula species from western Amazonia (Araneae, Theraphosidae, Theraphosinae)

**DOI:** 10.3897/zookeys.448.7920

**Published:** 2014-10-20

**Authors:** Fernando Pérez-Miles, Alexandre Bragio Bonaldo, Laura Tavares Miglio

**Affiliations:** 1 Sección Entomología, Facultad de Ciencias, Iguá 4225, 11400 Montevideo, Uruguay; 2 Laboratório de Aracnologia, Museu Paraense Emílio Goeldi, C.P. 399, 66017-970 Belém, Pará, Brazil

**Keywords:** Tarantula, taxonomy, *Bumba*, *Maraca*, Amazônia, Caxiuanã

## Abstract

We propose the name *Bumba* as a new name for *Maraca*, preoccupied by *Maraca* Hebard, 1926 (Orthoptera). We describe and illustrate *Bumba lennoni*, a new theraphosid species from Caxiuanã, Pará, Brazil. This species differs from the other species of the genus in the extremely reduced keel on male palpal organ and in the higher number of labial and maxillary cuspules. Females additionally differ in the spermathecal morphology. As a consequence of the name replacement, three new combinations are established.

## Introduction

The genus *Maraca* Pérez-Miles, 2005 was originally described as a replacement name for *Iracema* Pérez-Miles, 2000 which was preoccupied by *Iracema* Triques, 1996 in Pisces. Pérez-Miles (2005) was again unaware that the name *Maraca* was previously used for a Neotropical cockroach (Hebard 1926). To remove this generic homonymy the name *Bumba* is here proposed for *Maraca* Pérez-Miles (2005). The type species *Bumba cabocla* (Pérez-Miles 2000), comb. n. is so far recorded solely for the type locality, Maracá Island, State of Roraima northern Brazilian Amazonia. *Bumba horrida* (Schmidt 1994), comb. n. was transferred from the genus *Paraphysa* Simon, 1892 by Bertani and Carla-da-Silva (2003) to *Iracema* and, by the homonymy, fall into *Maraca* and now into *Bumba*. These authors extended the records of *Bumba horrida* from its type locality in Amazonas Federal Territory, Venezuela to the states of Amazonas and Roraima, Brazil. Recently, Perafán and Pérez-Miles (2014) transferred *Euathlus pulcherrimaklaasi* (Schmidt, 1991) to *Maraca* including Ecuador in the distribution of the genus.

*Bumba* (formerly *Maraca*) is characterized by the presence of type IV urticating hairs, a retrolateral process on male palpal tibiae, palpal bulb resting in a ventral distal excavation of palpal tibia, male metatatarsus I passing between tibial spurs when flexed, and spiniform setae on prolateral and retrolateral maxillae and coxae I–IV (Pérez-Miles 2000, Bertani and Carla-da-Silva 2003). Besides these characters, the reduced number of cuspules on labium was also indicated as diagnostic for *Bumba*; the new species fits in all other generic characters but has about 50 cuspules on labium. Consequently, the diagnosis of *Bumba* is presently modified in this point. We here diagnose, describe and illustrate *Bumba lennoni* sp. n., based on male and female specimens from Caxiuanã, Pará, Brazil. Some brief additional natural history comments on *Bumba lennoni* sp. n. are given. Three new combinations are established: *Bumba cabocla* (Pérez-Miles, 2000), *Bumba horrida* (Schmidt 1994) and *Bumba pulcherrimaklaasi* (Schmidt, 1991).

## Methods

Abbreviations: AME = anterior median eyes, ALE = anterior lateral eyes, PME = posterior median eyes, PLE = posterior lateral eyes, OQ = ocular quadrangle (including lateral eyes), d = dorsal, p = prolateral, r = retrolateral, v = ventral; MPEG = Museu Paraense Emílio Goeldi (Belém, Pará, Brazil). Male palpal organ keel terminology (following Bertani 2000): PSK = prolateral superior keel; PIK = prolateral inferior keel; SAK = subapical keel. All measurements are in millimeters (mm) and were taken using an ocular micrometer. The total length excludes chelicerae and spinnerets. Drawings were made with a camera lucida, with a stereomicroscope Zeiss Discovery V8. Urticating setae terminology follows Cooke et al. (1972). The number of cuticular extensions in paired appendages are expressed as right/left.

## Taxonomy

### Genus Bumba

Taxon classificationAnimaliaAraneaeTheraphosidae

Pérez-Miles, Bonaldo & Miglio, 2014

Iracema Pérez-Miles, 2000: 141 (pre-occupied, nec *Iracema* Trique, 1996).
Maraca Pérez-Miles, 2005: 247 (pre-occupied, nec *Maraca* Hebard, 1926).

#### Diagnosis.

*Bumba* differs from other genera of Theraphosinae in the combined presence of type IV urticating hairs, retrolateral process in male palpal tibiae, palpal bulb resting in a ventral distal excavation of palpal tibia, male metatatarsus I passing between tibial spurs when flexed, and spiniform setae on prolateral and retrolateral maxillae and coxae I–IV.

#### Type species.

*Bumba cabocla* (Pérez-Miles, 2000), comb. n.

#### Etymology.

Bumba (feminine) is taken from Brazilian theatrical folk tradition of the popular festival called Boi-bumbá (hit my bull), which takes place annually in North and Northeastern Brazil.

#### New combinations.

*Bumba cabocla* (Pérez-Miles, 2000), comb. n.; *Bumba horrida* (Schmidt, 1994), comb. n.; and *Bumba pulcherrimaklaasi* (Schmidt, 1991), comb. n.

### Bumba lennoni sp. n.

Taxon classificationAnimaliaAraneaeTheraphosidae

http://zoobank.org/382133FC-96E8-44CC-A4C3-7B0395671910

#### Type material.

Holotype ♂, 01°44'18.02"S, 51°27'48.01"W (DMS), Estação Científica Ferreira Penna, FLONA Caxiuanã, Melgaço, Pará, Brazil, 02.VI.2003, J. A. P. Barreiros & C. O. Araújo leg. (MPEG 983). **Paratypes:** BRAZIL. *Pará*: Melgaço, FLONA Caxiuanã, Estação Científica Ferreira Penna, 01°43'43.2"S, 51°29'00.7"W (DMS), Plot TEAM 2, 2♀♀, 03.X.2005, B. C. Araújo leg. (MPEG 19040); 05.X.2005, N. Abrahim leg. (MPEG 19041); 01°44'18.02"S, 51°27'48.01"W (DMS), 4♂♂, 10.VII.2002, D. E. Guimarães leg. (MPEG 1001); 19.VIII.2003, J. A. P. Barreiros leg. (MPEG 976); 24.VIII.2003, J. A. P. Barreiros leg. (MPEG 985); 19.X.2003, J. A. P. Barreiros & L. T. Miglio leg. (MPEG 975); 1♀, 21–30.XI.2000, A. B. Bonaldo leg. (MPEG 1924); 01°57'38.9"S, 51°36'45.3"W (DMS), Acampamento PPBio, Plot PPBio, 1♀, 10.V.2005, C. A. Lopes leg. (MPEG 19039). All deposited at MPEG.

#### Diagnosis.

Differ from the other species of *Bumba* in the very reduced keel on male palpal bulb and the higher number of labial cuspules (nearly 50).

#### Description.

**Figures 1–9.**
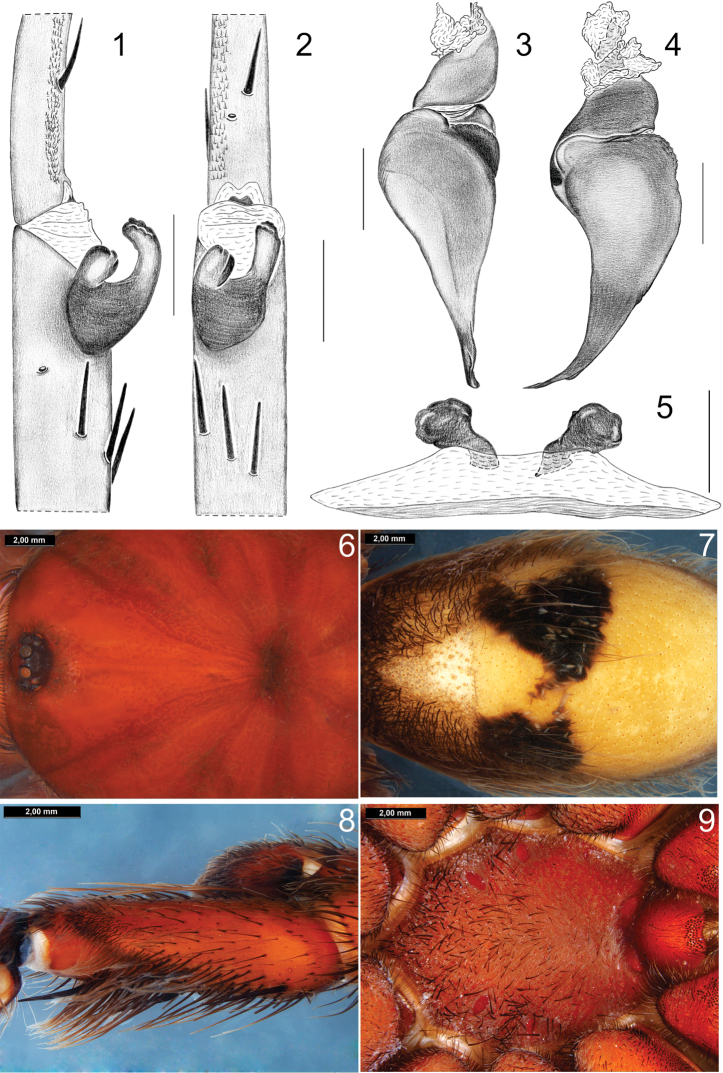
*Bumba lennoni* sp. n.: **1–4** and **6–8** male holotype (MPEG 983) **5, 9** female paratype (MPEG 19039). **1–2** Tibiae and metatarsi of left leg I: **1** Prolateral **2** Ventral **3–4** Copulatory bulb: **3** Prolateral **4** Retrolateral **5** Spermathecae, dorsal **6** carapace, dorsal **7** abdomen, dorsal **8** retrolateral process in male palpal tibiae **9** sternum, ventral Scales: **1–2:** 3 mm; and **3–5:** 1 mm.

**Figures 10–15.**
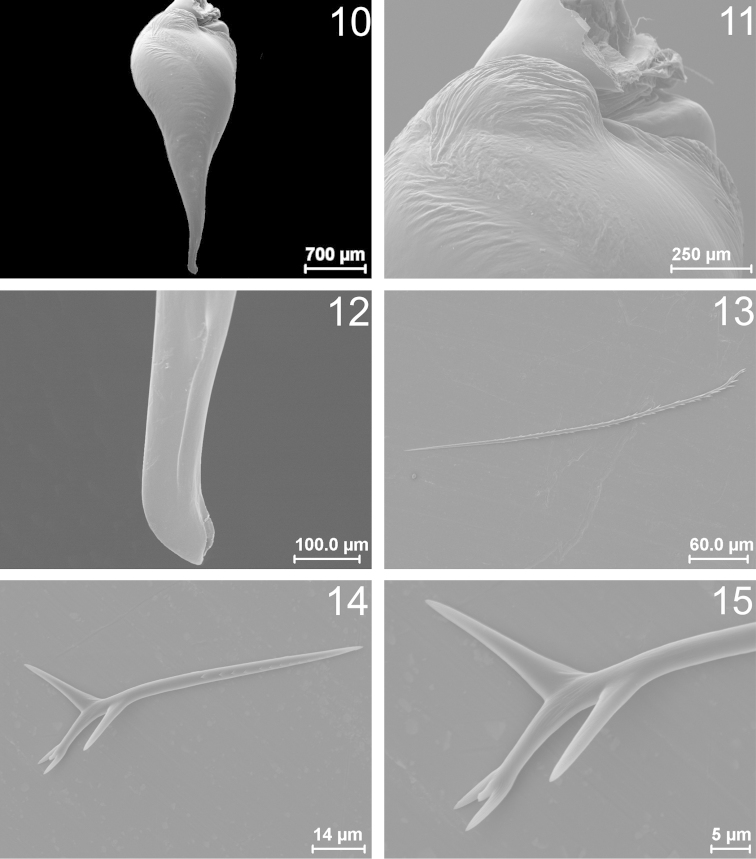
*Bumba lennoni* sp. n.: **10–13** male paratype (MPEG 975) **14–15** female paratype (MPEG 19039). **10–12** Copulatory bulb: **10** Prolateral **11** Prolateral, tegular apophysis (TA), detail **12** Prolateral, ring-shaped keel, detail **13** Type III urticating hair, silhouette **14–15** Type IV urticating hair: **14** Silhouette; **15** Tip, detail.

Male (holotype, MPEG 983): Total length 34.00, carapace length 17.50, width 14.75 Anterior eye row procurved, posterior recurved. Eyes sizes and interdistances: AME 0.42, ALE 0.66, PME 0.38, PLE 0.42, AME-AME 0.42, AME-ALE 0.36, PME-PME 1.32, PME-PLE 0.24 ALE-PLE 0.18, OQ length 0.98, width 2.94, clypeus 0.56. Fovea transverse, straight, width 2.06. Labium length 1.90, width 2.80, with 58 cuspules, maxillae with 218 cuspules in a triangular group with base on the proximal edge. Sternum length 7.75, width 6.63, posterior sigillae submarginal. Chelicerae with 12/11 promarginal teeth (5 to 8/9 from distal tip, smaller); a group 29/30 very small proximal teeth, behind promarginal ones. Tarsi I–IV densely scopulate, scopula I–III entire, IV divided by a narrow line of longer conical setae. Metatarsi I scopulate on distal half, II scopulate on distal third, III scopulate on distal fourth and IV apically scopulate. Tibia I with two prolatero-ventral, distal, unequal apophysis (Figs 1–2). Flexion of metatarsus I between both branches of the tibial apophysis. Palpal organ piriform (Figs 10–11), in Fig. 11 TA detailed; with distal ring-shaped keel (Fig. 12), perpendicular to major axis (Figs 3–4). Palpal tibia with a setose retrolateral process (Fig. 8); setae thick. Length of leg and palpal segments, in Table 1. Spination: Femora I, 3P; II, 3P, 1R; III, 4P, 3R; IV, 2P, 2R; palp, 1P. Patellae I, 1P, II, 0; III, 0; IV, 0; palp, 0. Tibiae I, 3P, 5R, 4V; II, 2P, 1R, 9V; III, 2P, 3R, 10V, 1D; IV, 5P, 9R, 12V, 2D; palp, 3P, 3V. Metatarsi I, 1P, 2R, 2V; II, 2P, 2R, 6V; III, 5P, 9R, 6V; IV, 6P, 8R, 14V; Tarsi I–IV and palp, 0. Color: Cephalothorax (Fig. 6) and legs reddish brown, abdomen light brown with central patch of urticating setae darker (Fig. 7). Type III (Fig. 13) and type IV urticating hairs present. PMS well developed, PLS normal, apical segment digitiform.

**Table 1.** Length of legs and palpal segments of holotype male *Bumba lennoni*.

	I	II	III	IV	Palp
Fe	16.13	15.00	13.25	16.50	7.88
Pa	8.75	8.63	6.88	7.25	5.25
Ti	13.63	12.50	10.25	13.63	6.88
Mt	13.50	13.38	15.88	21.13	-----
Ta	7.75	6.63	7.75	7.88	2.75
Total	59.76	56.14	54.01	66.39	22.76

**Variation** (range (mean ± standard deviation)): Total length 34.00–36.13 (35.20±0.93), carapace length 16.37–17.50 (17.02±0.51), width 14–15.25 (14.60±0.46). AME 0.40–0.64 (0.49±0.095), ALE 0.60–0.68 (0.64±0.04), PME 0.38–0.44 (0.40±0.02), PLE 0.40–0.44 (0.43±0.02), AME-AME 0.28–0.42 (0.33±0.06), AME-ALE 0.24–0.36 (0.30±0.06), PME-PME 1.08–1.32 (1.21±0.09), PME-PLE 0.04–0.24 (0.10±0.08), ALE-PLE 0.12–0.24 (0.19±0.04), OQ length 0.96–1.08 (1.00±0.046), width 2.20–2.94 (2.44±0.29), clypeus 0.56 (0.56±0.03). Fovea width 1.60–2.06 (1.93±0.31). Labium length 1.63–2.25 (1.91±0.22), width 1.75–2.80 (2.44±0.42).Sternum length 6.88–7.75 (7.25±0.34), width 5.5–6.63 (6.10±0.43). Legs: I 51.62–59.76 (56.22±2.47), II 49.63–56.14 (52.73±2.37), III 46.88–54.01 (49.80±2.62), IV 59.13–66.39 (62.53±2.69), palp 19.63–22.76 (21.58±1.22).

Female (Paratype, MPEG 19039): Total length 43.00, carapace length 20.38, width 16.63. Anterior eye row procurve, posterior row straight. Eye sizes and interdistances: AME 0.53, ALE 0.65, PME 0.44, PLE 0.56, AME-AME 0.44, AME-ALE 0.34, PME-PME 1.50, PME-PLE 0.19, ALE-PLE 0.25, OQ length 1.13, width 2.81, clypeus 0.84. Fovea width 2.28. Labium length 2.31, width 3.34, with 60 cuspules, maxillae with 264 cuspules. Sternum length 8.13, width 7.5, posterior sigillae submarginal (Fig. 9). Chelicerae with 14/13 promarginal teeth (from distal to proximal, 6/10 small, 7/6 to 13/10 medium sized); A basal group of 61/63 very small teeth, behind large ones. Tarsi densely scopulate, scopulae I–III entire, IV divided by a band of longer conical setae. Metatarsi I scopulate on distal half, II on distal third, III on apical fourth and IV without scopula. Length of leg and palpal segments in Table 2. Spination: Femora I, 2P; II, 1P; III, 1P,1D; IV, 1D; palp, 1P. Patellae I–II, 0, III, 1P; IV and palp, 0. Tibiae I, 1P, 5V; II, 1P, 5V; III, 2P, 2R,7V; IV, 2R, 5V; palp, 4P, 3V. Metatarsi: I, 3V; II, 1P, 5V; III, 2P, 6R,7V; IV, 2P, 4R, 11V. Tarsi I–IV and palp, 0. Color: Carapace and legs as in male, abdomen dark brown. Type III, IV (Figs 14–15) urticating hairs present, type IV modified, short with few (3–4) barbs. PMS slightly smaller than in male; PLS as in male. Spermathecae with two wide sub-spheric distal receptacles, very sclerotized, with a short neck with a narrow part sclerotized and a wide membranous area (Fig. 5).

**Table 2.** Length of legs and palpal segments of paratype female *Bumba lennoni*.

	I	II	III	IV	Palp
Fe	14.50	13.75	12.63	16.13	10.00
Pa	9.25	8.63	7.25	7.63	6.25
Ti	11.75	10.25	9.13	12.00	7.75
Mt	9.50	9.63	12.88	19.00	-----
Ta	6.00	5.38	5.75	6.00	6.13
Total	51	47.64	47.64	60.76	30.13

**Variation** (range (mean ± standard deviation)): Total length 26.25–43.00 (31.50±7.80), carapace length 16.50–20.38 (18.00±1.68), width 13.37–16.63 (14.72±1.41). AME 0.40–0.56 (0.48±0.08), ALE 0.60–0.72 (0.66±0.05), PME 0.36–0.44 (0.40±0.03), PLE 0.56 (0.46±0.07), AME-AME 0.24–0.44 (0.34±0.10), AME-ALE 0.34 (0.29±0.10), PME-PME 1.50 (1.22±0.21), PME-PLE 0.19 (0.77±0.49), ALE-PLE 0.25 (0.22±0.03), OQ length 1.00–1.13 (1.08±0.06), width 2.16–2.81 (2.42±0.28), clypeus 0.56–0.84 (0.69±0.12). Fovea width 1.92–2.56 (2.24±0.26). Labium length 1.75–2.31 (2.05±0.27), width 2.50–3.34 (2.71±0.43). Sternum length 6.88–8.13 (7.32±0.59), width 6.38–7.5 (6.75±0.51). Legs: I 38.25–51.00 (42.50±5.95), II 35.25–47.64 (40.94±5.30), III 35.88–47.64 (40.76±5.23), IV 46.63–60.76 (52.50±6.29), palp 24.50–30.13 (26.94±2.53).

#### Etymology.

The specific name is patronymic in honor of John Winston Lennon (1940–1980), the legendary creator of The Beatles, who contributed to make this world a gentler place.

#### Natural history.

All specimens from Estação Científica Ferreira Penna, FLONA Caxiuanã were collected in pit-fall traps used for herpetological surveys or in nocturnal manual searching, in both flooded and dry areas.

## Supplementary Material

XML Treatment for Bumba

XML Treatment for Bumba lennoni
